# Isometric vs Isotonic Core Stabilization Exercises to Improve Pain and Disability in Patients with Non-specific Chronic Low Back Pain: A Randomized Controlled Trial

**DOI:** 10.5812/aapm-144046

**Published:** 2024-02-15

**Authors:** Arash Khaledi, Mehdi Gheitasi

**Affiliations:** 1 Department of Sports Science, Kish International Campus, University of Tehran, Kish, Iran; 2 Department of Health & Sport Rehabilitation, Faculty of Sport Science & Health, University of Shahid Beheshti, Tehran, Iran

**Keywords:** Nonspecific Chronic Low Back, Exercise Therapy, Core Stability, Isometric, Isotonic

## Abstract

**Background:**

Non-specific chronic low back pain (NSCLBP) is a prevalent condition that affects 90% of individuals experiencing low back pain. Core stabilization exercises (CSE) stand out as the most commonly employed therapeutic approach for managing NSCLBP. Nevertheless, there remains uncertainty regarding the superior effectiveness between isometric (ISOM) and isotonic (ISOT) types of CSE in the treatment of NSCLBP.

**Objectives:**

The primary objective of this study was to compare the efficacy of ISOM and ISOT exercises concerning pain and disability in patients with NSCLBP. Additionally, the study aimed to assess the effectiveness of both ISOM and ISOT in comparison to no intervention concerning these variables in these patients.

**Methods:**

This study was a randomized controlled trial that involved 41 men and women experiencing NSCLBP. Participants were randomly allocated to three groups: ISOM CSE (n = 13), ISOT CSE (n = 14), and a waitlist control (n = 14). The exercise training was administered for 40 - 60 minutes three times a week over a period of up to 8 weeks. Pain (assessed using the Visual Analog Scale or VAS) and disability (evaluated through the Oswestry Disability Index or ODI) variables were measured before and after the interventions.

**Results:**

Based on the results, there was no significant difference between the 2 exercise groups (ISOM and ISOT) regarding pain and disability. However, the ISOM group demonstrated numerically better results than the ISOT group. Both the ISOM and ISOT groups exhibited a significant decrease in pain levels, with the VAS score decreasing from 5.5 to 2.7 for ISOM and from 5.8 to 3.7 for ISOT, as compared to the control group (P < 0.001 and P = 0.001, respectively). Additionally, the average disability showed a significant improvement in both the ISOM (ODI score from 17 to 11) and ISOT (ODI score from 15.4 to 11) groups compared to the control group (P < 0.001).

**Conclusions:**

Both ISOM and ISOT methods are effective in alleviating pain and disability in patients with NSCLBP. However, there is no significant difference in the benefits between them. Numerically, ISOM exercises were found to be superior. Further studies are needed to obtain a more accurate answer regarding their superiority.

## 1. Background

Low back pain is the second most common reason for visiting a doctor after a cold (1). Approximately 80% of people experience it at least once in their lifetime (2). The rate of disability due to low back pain has increased by 54% from 1990 to 2015 (3). Almost 10% of patients with low back pain have specific symptoms with a known cause, such as spondylolisthesis, lumbar spinal stenosis, fracture of the spine, nerve root compression, or inflammatory disease (2). However, 90% of these patients are diagnosed without specific symptoms, known as non-specific chronic low back pain (NSCLBP), where the cause of the disease cannot be identified clinically (4). Non-specific chronic low back pain (NSCLBP) persists for at least three months, and its occurrence area is beneath the costal margin and above the inferior gluteal folds without leg pain (5).

The most crucial non-surgical methods for treating patients with NSCLBP include pharmacotherapy (NSAID, muscle relaxants, opioids, glucocorticoids, etc.) (6), physical therapy (TENS, ultrasound, and hot packs), and exercise therapy (5). However, pharmacotherapy may lead to side effects such as constipation, nausea, and fatigue, and prolonged use of these drugs may result in a resurgence of pain upon discontinuation (7). On the other hand, physiotherapy without the incorporation of therapeutic exercise is not suitable for alleviating long-term pain and enhancing performance because it is relatively expensive and lacks active muscle contraction (8). Therefore, exercise therapy emerges as a more effective and affordable option for therapists and specialists in treating patients with NSCLBP, given its minimal harm and ease of implementation (5).

In 1992, Panjabi proposed a theory concerning the neutral zone of the spine and instability. He suggested that defects or weaknesses in any of the active spinal muscles (i.e., muscles and soft tissue), passive spinal column (i.e., vertebrae and skeletal structure), or neural subsystems can lead to defects in stability, low-back problems, and pain (9). Since then, therapists and researchers worldwide have developed therapeutic exercises under the umbrella term of core-based exercises, such as yoga, pilates, and core stabilization exercises (CSE), aiming to enhance stability and alleviate low-back problems like chronic pain, disability, and poor quality of life (9, 10). Recently, 4 systematic review and meta-analysis studies have also emphasized core-based exercises as the most effective therapeutic method for treating NSCLBP (8, 10-12).

Core stabilization exercises methods have gained more attention among core-based exercises. According to 2 systematic reviews (13) and a meta-analysis (14), CSE methods have proven more effective than other exercise approaches in reducing pain and improving disability. These exercises concentrate on activating and strengthening the muscles in the core region of the body, such as the paraspinal, multifidus, oblique and anterior abdominal, and gluteal muscles, through the performance of isometric (ISOM) and isotonic (ISOT) exercises (15).

Isometric core stabilization exercises (ISOM CSE) involve maintaining static positions for extended periods (16). These exercises primarily engage deep, slow-twitch, postural muscles (tonic muscles) responsible for maintaining good posture and supporting the body against gravity (16, 17). These muscles have a tendency to shorten over time, contributing to poor posture and back pain. Examples of these muscles include the paraspinal muscles, multifidus, transversus abdominis, and diaphragm, all located in the core area of the body (17, 18).

Isotonic CSE, or ISOT concentric and eccentric contractions involve dynamic movements that lead to both stretching and shortening of the muscles engaged in the exercise (16). In contrast to ISOM or ISOM contractions, ISOT CSE primarily engages phasic muscles, which are large and fast-twitch muscles located on the surface of the body (17). However, these muscles have a tendency to weaken and tire quickly. Examples of phasic muscles include the erector spinae, latissimus dorsi, rectus abdominis, and gluteal muscles (17, 18).

Despite extensive research in this area (8, 10-12, 14), the effectiveness of ISOM or ISOT exercises for patients with NSCLBP is still unclear. Previous studies either did not report the effectiveness of these 2 types of exercises separately or examined their combined effect (5, 16-18). Therefore, it remains unknown which type of exercise is more effective (19, 20). To our knowledge, there is no randomized controlled trial comparing ISOM and ISOT CSE without combining them with other interventions, such as physiotherapy, on pain and disability in patients with NSCLBP. Additionally, the latest review and meta-analysis did not report any strong evidence regarding the effectiveness of core exercises. Therefore, the study suggests conducting more randomized controlled trials in the future (21). This research aims to address the gaps in existing knowledge related to the pain and disability experienced by patients with NSCLBP.

## 2. Objectives

The main objective of this study was to compare the efficacy of 2 different CSE methods, ISOM and ISOT, on pain and disability in patients with NSCLBP. Additionally, the study aimed to assess the effectiveness of each of the 2 ISOM and ISOT methods compared to no intervention (control group) on the same variables in these patients.

The primary hypothesis of the study is that both CSE methods (ISOM and ISOT) are more effective than no intervention in reducing pain and disability in patients with NSCLBP (14). The second hypothesis posits that there is no significant difference in the effectiveness of these two exercise methods on pain (13). Lastly, considering the dynamic nature of ISOT exercises, it is hypothesized that they are more effective in improving the disability of these patients compared to ISOM (22).

## 3. Methods

### 3.1. Study Design

This study was a randomized controlled trial conducted between May and November 2023 at the sports rehabilitation laboratory of Shahid Beheshti University in Tehran, Iran. Despite an omission error, the trial protocol was retrospectively registered on May 1, 2023, in the Iranian Registry of Clinical Trials (IRCT Identifier: IRCT20180727040609N2). Before initiating the research, informed consent was obtained from all participants. The study was conducted in accordance with the Declaration of Helsinki (23) and received approval from the Ethics Committee of the Sports Sciences Research Institute in Tehran, Iran (approval ID: IR.SSRC.REC.1401.155).

### 3.2. Participants

**Figure 1.**
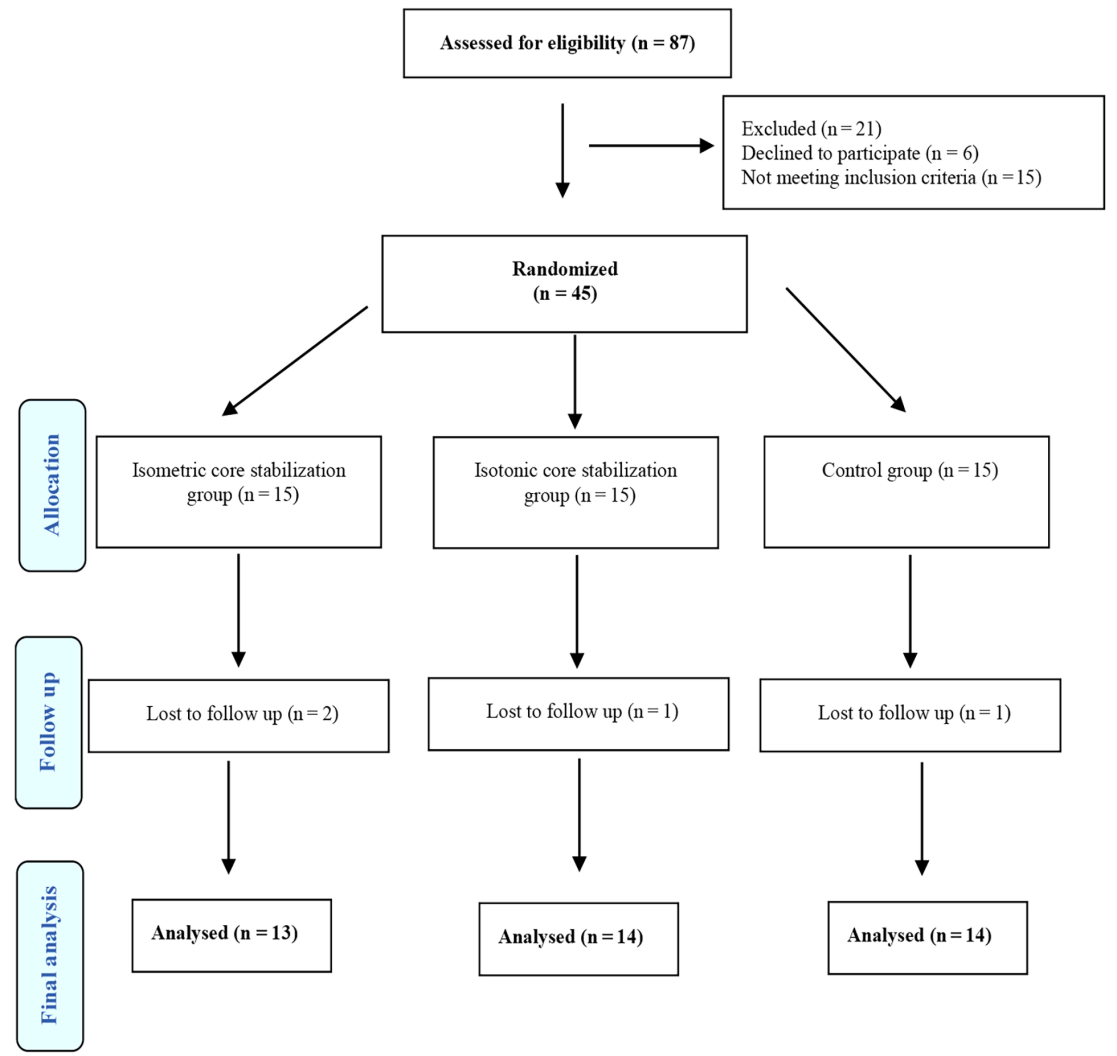
Flow diagram of the participants.

The study involved 41 patients diagnosed with NSCLBP by a specialist who subsequently prescribed exercise therapy. The patients were divided into 3 groups: ISOM CSE (n = 13), ISOT CSE (n = 14), and control (n = 14). The groups comprised 20 women and 21 men aged between 22 and 56 years. To ensure fairness, a simple randomization method was employed to assign patients to their respective groups. Each participant selected a sealed envelope, and allocation was carried out using the “Research Randomizer" website (24). This method ensured concealment and a randomized cycle, thus preventing any bias in the selection process (25). Researchers used G*Power 3.1 to calculate the minimum sample size for their study, employing an alpha value of 0.05, a power of 80%, and an effect size of 0.88 (26). The study required 10 participants in each group, but to account for possible dropouts, 15 patients were initially assigned to each group. During the study, three patients from the 2 experimental groups who did not attend the exercise sessions and one patient from the control group who did not attend the post-test were excluded (Figure 1). 

### 3.3. The Inclusion Criteria

The study's inclusion criteria required patients to meet the following conditions: they had to have experienced pain for more than three months, with a Visual Analog Scale or VAS score of 2 or higher. Additionally, participants needed to be between the ages of 20 and 60 and have a body mass index or BMI of 30 or less. Patients who had undergone any other treatments, such as acupuncture or physical therapy, in the last 3 months were not eligible for the study (5).

### 3.4. The Exclusion Criteria

The study excluded individuals who had low back pain due to other causes, previous spine surgery, fibromyalgia, spondylolisthesis or spondylolysis, rheumatoid arthritis or ankylosing spondylitis, spinal inflammation or tumor, spinal or pelvic fracture, osteoporosis, respiratory disease or heart disease, stroke, pregnancy (in women), or continuous use of pain medications. Participants who were non-compliant, attending less than 90% of sessions, or voluntarily withdrew from the study were also excluded (27).

### 3.5. Interventions

Each experimental group of patients underwent an 8-week therapeutic exercise program, with 3 sessions per week. Each session lasted between 40 and 60 minutes, including a warm-up period of 5 - 7 minutes, the main exercise period of 40 - 50 minutes, and a cool-down period of 3 - 5 minutes (5). The control group did not receive any intervention except for education. Following ethical principles, they were placed on an 8-week waiting list for treatment. During this period, they were observed. Following the 8-week intervention period, no follow-up assessments were conducted to determine whether the changes in patients were sustainable.

All three groups received education. To facilitate this, each patient received an educational pamphlet explaining how to maintain correct posture and use the abdominal brace method to avoid low back pain. The recommended practice was to employ light abdominal bracing, approximately 10% to 20% of the maximum bracing, at all times. The exercise groups were also provided with a separate pamphlet explaining how to perform the exercises correctly.

During the first visit, an educational session was conducted with the presence of an exercise therapy specialist. To ensure the correct implementation of the exercises, another training session was held in the presence of an expert. The exercises were continued at home until the end of the treatment period. Every 2 weeks, all patients were interviewed over the phone to confirm their current pain status, adjust their training level, and assess their exercise compliance.

**Figure 2.**
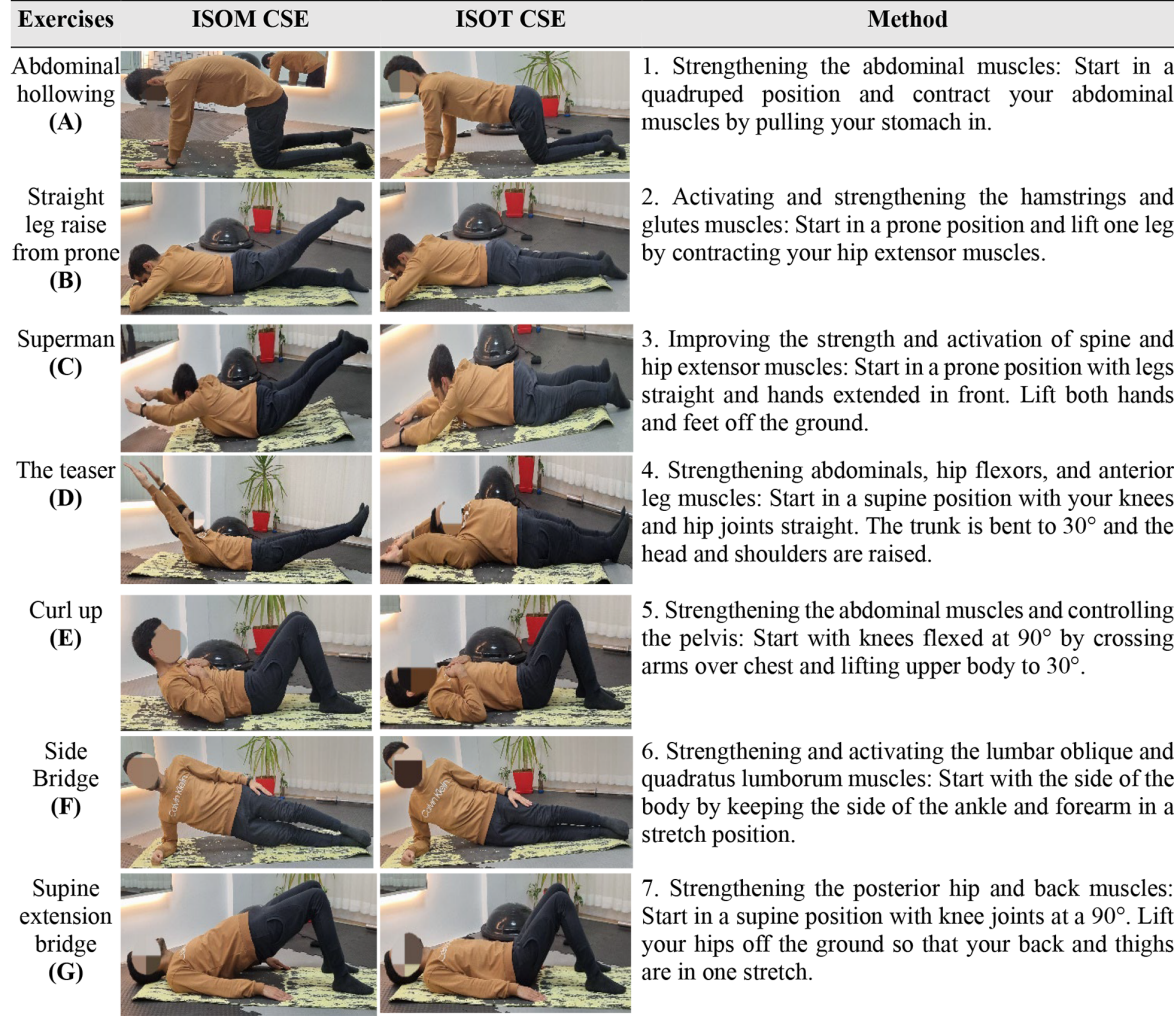
Method of isometric (ISOM) and isotonic (ISOT) core stabilization exercise (CSE). All exercises included 4 sets x 8 to 15 sec (for ISOM) and 4 sets x 6 to 12 reps (for ISOT).

It's important to note that the movements involved in ISOT and ISOM exercises are similar, except for the fact that ISOT exercises do not require sustaining a long-term contraction, whereas ISOM exercises require performing the same movements while maintaining a long contraction. The exercise routine (7 movements) includes the following: abdominal hollowing (A), straight leg raise from prone (B), superman (C), the teaser (D), curl up (E), side bridge (F), and supine extension bridge (G). Figure 2 provides more information regarding a set of exercises as described by Kisner (28).

### 3.6. Isometric Core Stabilization Exercises

During the ISOM exercises, patients were instructed to hold the contraction for 8 to 15 seconds, followed by a 5-second rest between each of the 4 sets. After each movement, patients were given a 1-minute rest before starting the next movement (Figure 2) (28). Throughout each repetition, patients were trained to contract their abdominal muscles and maintain the compression while sustaining a typical breathing pattern.

### 3.7. Isotonic Core Stabilization Exercises

The ISOT exercises were performed in 4 sets, with 6 to 12 repetitions in each set. Additionally, a 1-minute rest was applied between each movement (Figure 2). The exercises involved back-and-forth movements without any long pauses in muscle contraction (28).

### 3.8. Outcome Measures

#### 3.8.1. Measurement of Pain Intensity

The participants' level of pain was assessed using the visual analog scale (VAS). They were instructed to indicate their pain level on a 10-cm straight line with tick marks. A score between 0 and 10 was used as a criterion, where 0 indicates “no pain," and 10 indicates “the worst possible pain." The VAS measurement tool has high inter-rater reliability (intraclass correlation coefficient [ICC] = 1.00) and test-retest reliability (ICC = 0.99) in clinical studies (29).

#### 3.8.2. Measurement of Disability Level

The Oswestry Disability Index (ODI) was employed to assess the level of disability in patients. It comprises 10 questions that evaluate different aspects of daily life, including pain intensity, personal care, lifting, walking, sitting, standing, sleeping, sexual activity, social life, and travel. Each question offers 6 response options, ranging from 0 (best performance) to 5 (worst performance) points. A higher score on the ODI reflects more severe disability (30). Both pain and disability were evaluated before and after 8 weeks.

### 3.9. Statistical Methods

The statistical analyses were conducted using SPSS (Version 22.0) for Windows (SPSS Inc., Chicago, IL, USA). The data are presented as mean ± SD in the table and text. Data were checked for normal distribution. To identify differences between groups, one-way ANOVA and ANCOVA were utilized at the significance level of P ≤ 0.05.

## 4. Results

Kurtosis and Skewness for the variables were between -1 and +1, indicating the normal distribution of the data. The results of one-way ANOVA showed no significant differences between the individual characteristics and research variables of the subjects (P > 0.05), demonstrating homogeneity of the subjects in the respective randomized groups.

**Figure 3.**
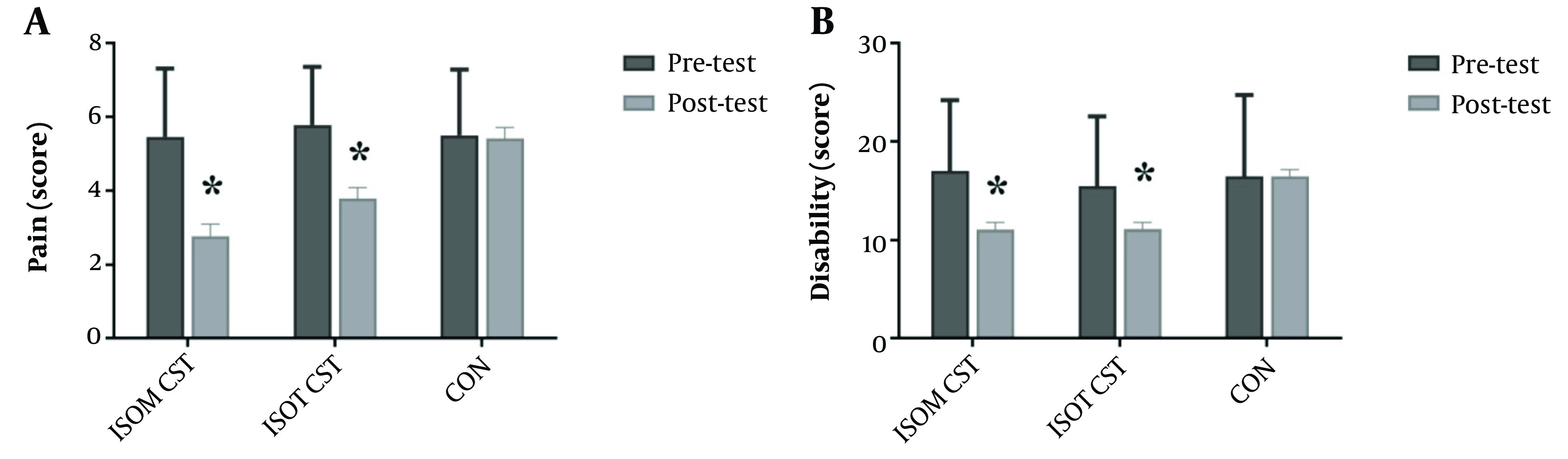
The results of bonferroni post-hoc test (* P ≤ 0.05, A significant difference with the control group).

The results of the covariance analysis for the VAS test (pain intensity) indicate a significant difference among the three groups: ISOM, ISOT, and CON (P < 0.001, η2 = 0.51 and F37, 2 = 19.5) as shown in Table 1. The Bonferroni post hoc test for comparing the means of pain intensity showed a significant difference between the CON group and the ISOM (P < 0.001) and ISOT groups (P = 0.001). Therefore, the pain intensity in the ISOM group (+2.6 ± 0.4) is lower than in the CON group, while the pain intensity in the ISOT group (+1.6 ± 0.4) is also lower than in the CON group (Table 2 and Figure 3). 

**Table 1.** Covariance Test Results for Group Comparison

Variables (Source)	SS	Df	MS	F	Sig	Effect Size
**Pain intensity**						
Pretest	57.2	1	57.2	46.3	< 0.001	0.56
Groups	48.1	2	24.1	19.5	< 0.001	0.51
Error	45.7	37	1.2	-	-	-
**Score disability level**						
Pretest	1352.1	1	1352.1	208	< 0.001	0.85
Groups	267	2	133.5	20.5	< 0.001	0.52
Error	240.5	37	6.5	-	-	-

Abbreviations: SS, sum-of-squares; df, degrees of freedom; MS, mean squares; F, F ratio.

**Table 2.** Individual Characteristics of the Subjects. Pre- and Post-pain and Disability Individual ^a^

Groups	ISOM CSE (F = 7, M = 6)	ISOT CSE (F = 6, M = 8)	CON (F = 7, M = 7)	Sig ^b^
**Age (y)**	40.4 ± 6.8	34.7 ± 6.9	36.8 ± 11.2	0.240
**Body height (cm)**	164.7 ± 10.5	174.3 ± 9.4	168.9 ± 12.6	0.086
**Body weight (kg)**	77 ± 9.7	77.6 ± 9.7	79.2 ± 9.2	0.820
**BMI (kg/m** ^**2**^ **)**	28.5 ± 3.7	25.5 ± 2.2	28 ± 3.9	0.057
**Pain (score)**				0.868 ^c^
Pre	5.5 ± 1.9	5.8 ± 1.6	5.5 ± 1.8	
Post	2.7 ± 1.6	3.7 ± 1.9	5.4 ± 1.7	
**Disability (score)**				0.862 ^c^
Pre	17 ± 7.2	15.4 ± 7.1	16.4 ± 8.3	
Post	11 ± 6.9	11 ± 7.3	16.4 ± 7.6	

Abbreviations: ISOM CSE, isometric core stabilization exercise; ISOT CSE, isotonic core stabilization exercise; F, female; M, male.

^a^ Values are presented as mean ± SD.

^b^ Significance level through one-way ANOVA test.

^c^Significant level in groups before intervention.

Similarly, the Oswestry Disability Index (score disability level) showed a significant difference among the three groups (ISOM, ISOT, and CON) (P < 0.001, η2 = 0.52 and F37, 2 = 20.5), as presented in Table 1. The Bonferroni post hoc test for comparing the means of the score disability level showed a significant difference between the CON group with the ISOM (P < 0.001) and ISOT groups (P < 0.001). As a result, the score disability level in the ISOM group (+5.4 ± 0.9) is lower than the CON group, while the score disability level in the ISOT group (+5.3 ± 0.9) is also lower than the CON group (Table 2 and Figure 3). 

## 5. Discussion

This study compared the effectiveness of 2 different CSE methods, isometric (ISOM) and isotonic (ISOT), in reducing pain and disability in patients with non-specific chronic low back pain (NSCLBP). After 8 weeks, the results showed that there was no significant difference between the 2 methods in terms of pain and disability improvement. However, the ISOM method exhibited slightly lower pain and disability scores compared to the ISOT method. Both the ISOM and ISOT exercise methods were found to be more effective in reducing pain and improving functional disability in patients with NSCLBP compared to receiving no intervention. The study provides valuable scientific insights, which will be discussed in detail.

The first null hypothesis of this research was that both CSE methods (ISOM and ISOT) are more effective than no intervention in reducing pain and improving disability in patients with NSCLBP (14). As expected, this study showed that both CSE methods were significantly more effective in reducing pain and improving disability compared to the non-intervention group. Meanwhile, the no-intervention group saw little to no change in their VAS score (from 5.5 to 5.4) and ODI score (from 16.4 to 16.4). These findings align with numerous original studies (5, 27, 31-33) and systematic review studies (10, 12-14). Recent systematic review and meta-analysis studies also support these results (10, 12, 34). Additionally, there was almost no disparity observed in the study (21).

Although the causes of NSCLBP are complex, many of them remain unknown (35). One of the most significant factors contributing to this disease is the weakness of the shallow trunk, abdominal, and gluteal muscles. Poor motor control in deep trunk muscles, such as transversus abdominis and multifidus, can also cause back pain (33). Core stabilization exercises can target both global and local muscle groups, providing stability to the lumbopelvic region, which can improve pain and disability in patients (10). The multifidus and transversus abdominis (local) muscles work together to stabilize the spine (17, 18). Other muscle groups (global) add stability by producing torque to counter external forces. When the core muscles work properly, they protect the spine and reduce stress on the lumbar vertebrae and intervertebral discs, which is why they are known as “the natural brace" (36).

Our second null hypothesis was that there is no significant difference between the efficiency of ISOT and ISOM methods in reducing pain (13). The findings of the research almost support this hypothesis as there was no statistically significant difference between the pain reduction achieved by ISOM (VAS score from 5.5 to 2.7) and ISOT (VAS score from 5.8 to 3.7) methods. However, numerically, the ISOM method showed a greater reduction in pain.

Hodges et al. pointed out the weakness and atrophic changes of intrinsic or deep (local) muscles in people with NSCLBP (36). These muscles are mainly tonic and involved in long-term muscle contractions (17, 36). Therefore, ISOM exercises are more likely to be effective in increasing the strength and endurance of these muscles compared to extrinsic or superficial (global) muscle groups (19, 36). This possible factor has reduced pain in these patients.

A recent systematic review and meta-analysis conducted by Sutanto et al. compared the effectiveness of motor control, ISOM, and ISOT trunk training interventions in reducing pain and disability in patients with NSCLBP. The authors found that trunk ISOM exercises were significantly more effective in improving pain and disability when compared to ISOT and motor control exercises (35). In the current study, there were no significant differences observed between the two exercise groups in the mentioned variables. This contrasts with the findings of Sutanto et al. The reason for this inconsistency could be that their systematic review and meta-analysis only considered articles related to the effectiveness of trunk training, whereas the present study included training of all core muscles (35).

The third hypothesis of this research assumed that due to the dynamic nature of ISOT exercises, it is more effective in improving patients' functional disability compared to ISOM exercises (22). However, the findings do not support this hypothesis, and the ISOM group still shows relatively better results (ODI score from 17 to 11) compared to the ISOT group (ODI score from 15.4 to 11), although this difference was not statistically significant. Interestingly, patients reported better functional status with ISOM training, contrary to expectations. It's worth noting that the training interventions in this study lasted for 8 weeks, and there is a possibility of significant changes in both pain and disability in the ISOM versus ISOT CSE with a longer therapeutic exercise period (e.g., 12 weeks).

According to Lederman's report, active-dynamic rehabilitation exercises are more effective than active-static ones as they activate sensory-motor systems, which are expected to improve functional disability following ISOT training (22). The results of the current research do not align with Lederman's report. One major reason for this could be that pain directly impacts an individual's performance, meaning that a reduction in pain can lead to better performance in daily activities (31). A recent study by Alarab et al. also showed similar findings to the present study. They compared ISOT and ISOM exercises and found no significant difference between the 2 methods in reducing pain and improving the disability of patients. These investigators used the common approach of physical therapy (TENS and infrared therapy) in their interventions, so the net effects of exercise alone were not determined. In addition, the role of ISOM exercises in the current research was more prominent, which may be inconsistent with their study. This difference could be due to the shorter treatment period of 4 weeks and the use of physiotherapy intervention (37).

It is possible that longer training periods (e.g., 12 weeks), more training sessions per week (e.g., 5 sessions), or manipulation of other factors such as volume, intensity, or duration of training could help in better deciding between ISOT and ISOM exercises. These gaps require further investigation in the future. Consequently, the difference in the benefits of the two exercises is still unclear. Future researchers are advised to consider and examine the above research gaps along with the limitations of this study, which will be discussed below. This will enable them to make a more informed decision when choosing the best exercise method for treating NSCLBP.

This study had certain limitations. Firstly, the patients examined only consisted of general individuals with NSCLBP between the ages of 22 and 56, and it was not possible to examine other groups, such as athletes and the elderly, which are of special importance. Secondly, only dependent variables of pain and disability were investigated, while other variables, such as fear of movement, quality of life, and muscle thickness, need to be studied as well. Additionally, it was not possible to assign more than 14 patients to each intervention group, and it was also not possible to blind therapists, assessors, and patients. Lastly, the treatment period was limited to a maximum of 8 weeks.

This study also has many strengths. It was a randomized controlled trial, considered one of the most reliable forms of clinical research, and included both a control group and a follow-up period. This study compared 2 CSE methods, ISOM and ISOT, without combining them with other therapeutic interventions such as physiotherapy or acupuncture. This allowed us to detect the net effects of each of these training methods for the first time. Furthermore, the study used CSE, which can be easily performed without the use of special equipment.

### 5.1. Conclusions

The study results indicate that both CSE methods (ISOM and ISOT) are effective in reducing pain and improving disability in patients with NSCLBP when compared to no intervention. Although no significant difference was found between the effectiveness of ISOM and ISOT exercises, numerically, ISOM exercises were observed to be more beneficial. Therefore, more randomized controlled trials with a larger cohort of patients and a training period exceeding 8 weeks are needed to conclusively determine the superiority of one of these 2 CSE methods in the future.

## Data Availability

The corresponding author can provide the dataset upon request during or after publication.

## Clinical Trial Registration Code:

The Iranian Registry of Clinical Trials (IRCT Identifier: IRCT20180727040609N2).
